# A bedr way of genomic interval processing

**DOI:** 10.1186/s13029-016-0059-5

**Published:** 2016-12-15

**Authors:** Syed Haider, Daryl Waggott, Emilie Lalonde, Clement Fung, Fei-Fei Liu, Paul C. Boutros

**Affiliations:** 1 Informatics and Biocomputing Platform, Ontario Institute for Cancer Research, Toronto, M5G 0A3 Canada; 2 Departments of Radiation Oncology, Pharmacology & Toxicology, and Medical Biophysics, University of Toronto, Toronto, M5G 2M9 Canada; 3 Ontario Cancer Institute and Campbell Family Institute for Cancer Research, Princess Margaret Hospital, University Health Network, Toronto, M5G 2M9 Canada

**Keywords:** Genomic intervals, BED format, Sequence algebra, Data integration

## Abstract

**Background:**

Next-generation sequencing is making it critical to robustly and rapidly handle genomic ranges within standard pipelines. Standard use-cases include annotating sequence ranges with gene or other genomic annotation, merging multiple experiments together and subsequently quantifying and visualizing the overlap. The most widely-used tools for these tasks work at the command-line (e.g. BEDTools) and the small number of available R packages are either slow or have distinct semantics and features from command-line interfaces.

**Results:**

To provide a robust R-based interface to standard command-line tools for genomic coordinate manipulation, we created bedr. This open-source R package can use either BEDTools or BEDOPS as a back-end and performs data-manipulation extremely quickly, creating R data structures that can be readily interfaced with existing computational pipelines. It includes data-visualization capabilities and a number of data-access functions that interface with standard databases like UCSC and COSMIC.

**Conclusions:**

bedr package provides an open source solution to enable genomic interval data manipulation and restructuring in R programming language which is commonly used in bioinformatics, and therefore would be useful to bioinformaticians and genomic researchers.

**Electronic supplementary material:**

The online version of this article (doi:10.1186/s13029-016-0059-5) contains supplementary material, which is available to authorized users.

## Background

With the advent of high-throughput sequencing technologies, data scientists are facing immense challenges in large-scale sequence analysis and in integrating genomic annotations. For instance, comparing new experiments with previously published datasets, translating genomic coordinates between different assemblies of an organism as well as finding cross-species orthologues are some of the common use-cases in basic science experiments. To assist these tasks genomic features are routinely represented and shared using Browser Extensible Display (BED; [1]), Distributed Annotation System (DAS; [2]), General Feature Format (GFF), Gene Transfer Format (GTF) and Variant Call Format (VCF). These all enable cross-sectional analysis of genomic studies across multiple programming languages, thereby enabling seamless data-integration.

R is the *de facto* standard for statistical analysis and visualization in computational biology [3] for both exploratory prototyping and rigorous production pipelines. To this end R has adopted several packages, such as GenomicRanges and IRanges that expressly deal with genomic intervals [4]. Albeit powerful, these existing tools require understanding of bespoke data structures and classes/objects. To address these issues we implemented a formal BED-operations framework called bedr, which is an R API offering utility functions implementing commonly used genomic operations as well as offering a formal R interface to interact with BEDTools and BEDOPS. bedr is fully compatible with the ubiquitous BED tools [5, 6] paradigm and integrates seamlessly with R-based work-flows.

## Implementation

**Fig. 1:**
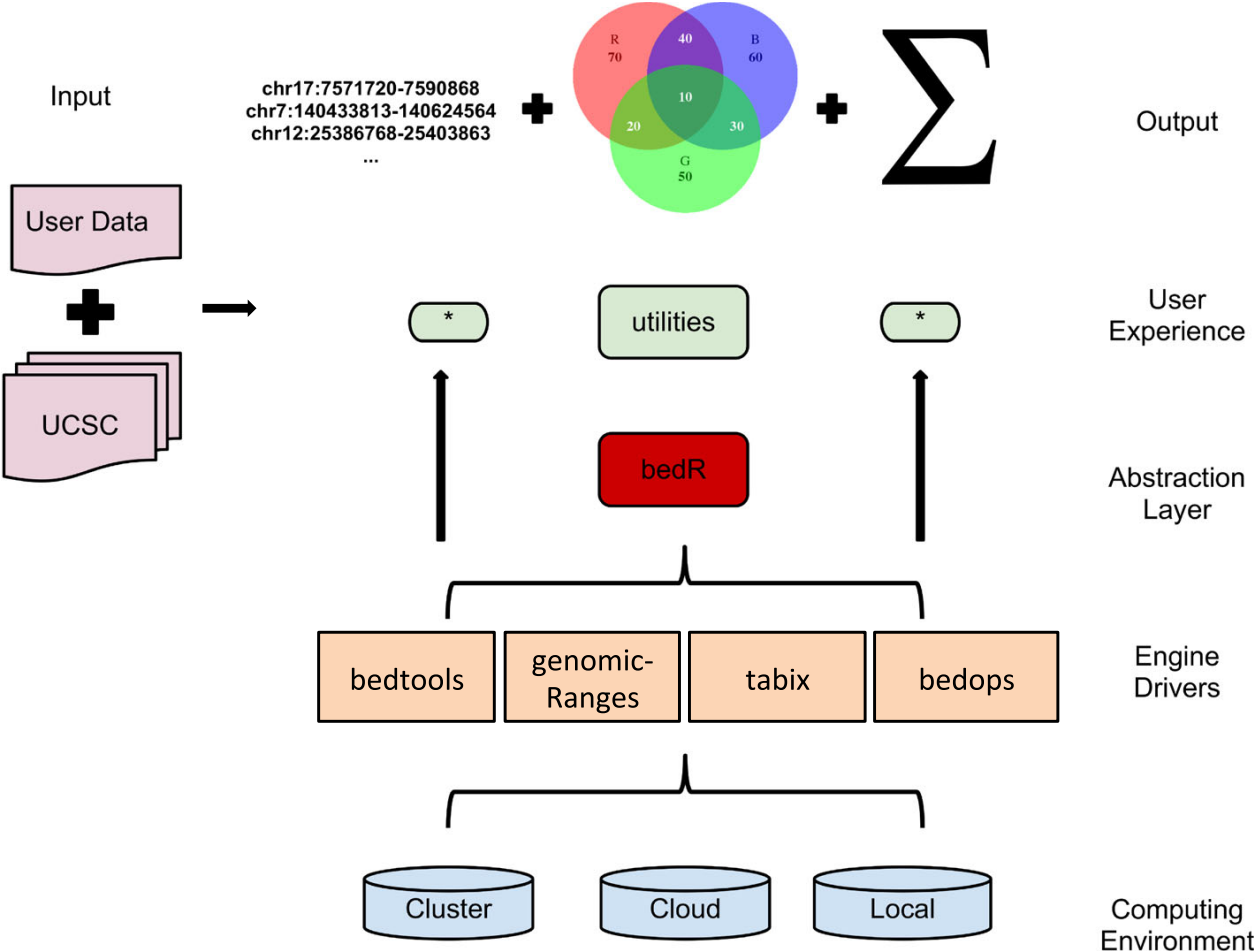
Overview of bedr package. bedr can run on a commodity linux based computer or a cloud/cluster. Users can interface with the underlying driver engines such as BEDTools/BEDOPS/tabix/GenomicRanges through bedr methods in R. This enables integration of user-specified multiple genomic intervals with reference data sources such as gene annotations (e.g. UCSC) and disease specific features (e.g. COSMIC). Such integration spans general-purpose genomic interval operations of intersection (*), union (sum) and joins. Output is returned in R friendly data structures for convenience in subsequent downstream analyses. These data structures are readily convertible to standard data exchange formats such as *BED* and *GRanges* using bedr utility methods

bedr is implemented in R and supports the two main BED engines: BEDTools and BEDOPS [5, 6]. It works on Unix-like operating systems including Mac OSX. A high-level conceptual overview of the package usage is shown in Fig. 1. bedr functions run on any computing platform (commodity computer, cluster or cloud), and facilitates integration of multiple data sources (e.g. gene annotations) and tools (e.g. BEDTools) with user-specified genomic coordinates, yielding fast results in R data structures such as *data.frame* and *list*. All API calls are written in R, which internally calls BEDTools and/or BEDOPS utilities in the native format using system commands. Further, bedr data can be readily converted to *BED* [1] and *GRanges* [4] objects for wider interoperability.

## Results and discussion

The primary input to most bedr methods is a regions object, which is represented as either an R *vector* of multiple region strings as illustrated below or a *data.frame* of regions with three columns: *chr, start,* and *end*. The regions object returned by various bedr methods matches the input format; *vector* or *data.frame*. Here we briefly summarize a subset of key bedr functionalities. For further details on a range of bedr utilities, please see package’s help and vignettes for detailed examples and workflows.

### Sort & merge

This functionality enables sorting of genomic regions in both natural and lexographical order using R, unix, BEDTools and BEDOPS engines. Following examples demonstrate the usage of these engines:

- regions <- get.example.regions()
- region <- regions[[1]]
- bedr.sort.region(
  - x = region,
  - engine = "unix",
  - method = "natural"
  - )
- bedr.sort.region(
  - x = region,
  - engine = "R",
  - method = "lexicographical"
  - )
- bedr.sort.region(
  - x = region,
  - engine = "bedtools"
  - )
- bedr.sort.region(
  - x = region,
  - engine = "bedops"
  - )

The above code will generate the following outputs of sorted regions:

- # natural sort (unix)
- "chr1:10-100" "chr1:101-200"
- "chr1:200-210" "chr1:211-212"
- "chr2:10-50" "chr2:40-60"
- "chr10:50-100" "chr20:1-5"

- # lexicographical sort (R)
- "chr1:10-100" "chr1:101-200"
- "chr1:200-210" "chr1:211-212"
- "chr10:50-100" "chr2:10-50"
- "chr2:40-60" "chr20:1-5"

- # lexicographical sort (bedtools)
- "chr1:10-100" "chr1:101-200"
- "chr1:200-210" "chr1:211-212"
- "chr10:50-100" "chr2:10-50"
- "chr2:40-60" "chr20:1-5"

- # lexicographical sort (bedops)
- "chr1:10-100" "chr1:101-200"
- "chr1:200-210" "chr1:211-212"
- "chr10:50-100" "chr2:10-50"
- "chr2:40-60" "chr20:1-5"

As shown above, various types of sorting results are presented in a similar R data structures regardless of which sorting engine is used (unix, R, bedtools or bedops) and their respective output style. Also, BEDTools and BEDOPS do not support natural sorting, and if method = “natural” is requested with these two engines, bedr automatically defaults to using engine = “unix” of “R” to perform sorting. Note, sorting of large number of regions through R will be slow and may also result in high memory overhead.

Much of the command-line interaction with BEDTools and BEDOPS is performed through temporary files followed by efficient piping/parsing of the output straight into R data structures. This ensures that memory intensive sorting tasks (or any other genomic operations discussed below) are managed by the optimized engines such as (BEDTools or BEDOPS), and therefore memory operations in R are limited to subsequent parsing of output.

**Fig. 2:**
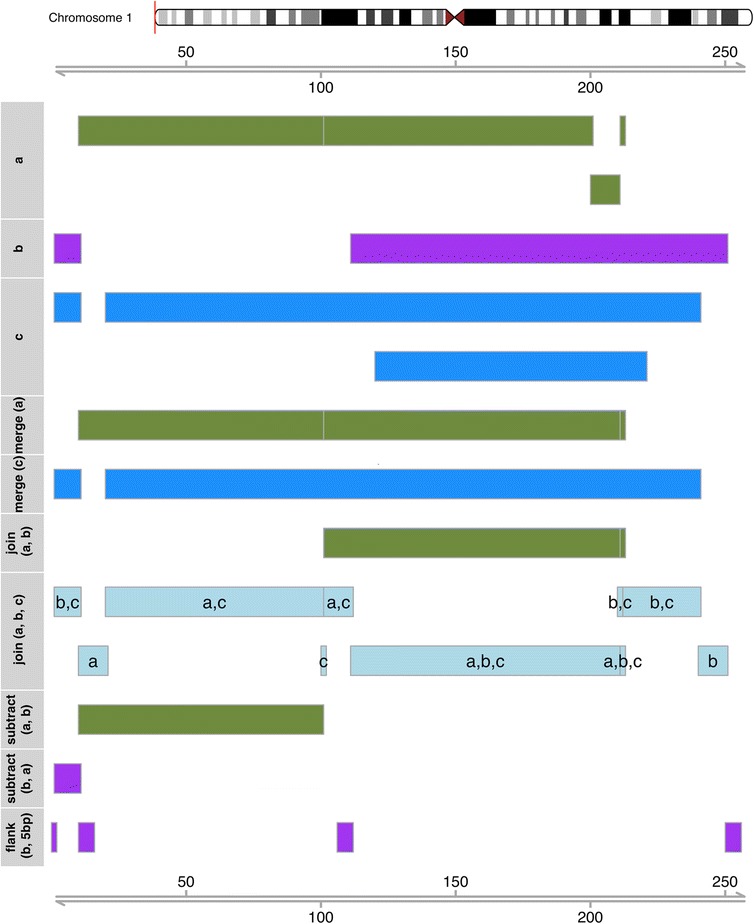
Illustration of key bedr operations. bedr regions objects represent a collection of sub-regions specified as R *vector* or *data.frame*. Three partially overlapping example regions (a, b and c) located at the beginning of human chromosome 1 (red mark on ideogram, 1-250 bp) are shown here. Vertical gray separators between sub-regions indicate regions that are 1 base pair apart. Overlapping regions can be merged, joined, subtracted resulting in new regions objects as shown here. Associated source code snippets are documented in the Results section. Regions object flank (b, 5 bp) exemplifies bedr utility *flank.regions* creating flanking (up and/or downstream) regions of a specified length; +/-5 bp in the example shown here

In addition to sort operations, bedr also supports identification of overlapping regions which can be collapsed to avoid downstream analytical challenges such as many:many join results (Fig. 2), e.g.

- bedr.merge.region(x = region)

The above code will generate the following output of merged regions:

- "chr1:10-100" "chr1:101-210"
- "chr1:211-212" "chr10:50-100"
- "chr2:10-60" "chr20:1-5"

Sort and merge can be combined into one step given they are generally run as a tandem preprocessing step:

- bedr.snm.region(x = region)

The above code will generate the following vector output of sorted and merged regions:

- "chr1:10-100" "chr1:101-210"
- "chr1:211-212" "chr10:50-100"
- "chr2:10-60" "chr20:1-5"

### Join

This functionality enables joining two region-based datasets using intervals as an index or primary key. The output is left outer join with respect to the first regions object (Fig. 2), e.g.

- regions.a <- bedr.merge.region(
  - x = regions[[1]]
  - )
- regions.b <- bedr.merge.region(
  - x = regions[[2]]
  - )
- regions.c <- bedr.merge.region(
  - x = regions[[4]]
  - )
- bedr.join.region(
  - x = regions.a,
  - y = regions.b
  - )

The above code will generate the following output, containing regions of regions.a in the first column, while any overlapping regions from regions.b are listed in columns 2 to 4 (chr, start, end). Regions in regions.a with no overlap are encoded as: . and -1

**Table:** 

	index	V4	V5	V6
1 2 3 4 5 6	chr1:10-100 chr1:101-210 chr1:211-212 chr10:50-100 chr2:10-60 chr20:1-5	. chr1 chr1 . chr2 .	-1 111 111 -1 40 -1	-1 250 250 -1 60 -1

Similarly, another bedr function bedr.join.multiple.region() supports merging of multiple sets of regions (Fig. 2), e.g.

- bedr.join.multiple.region(
  - x = list(
    - a = regions.a,
    - b = regions.b,
    - c = regions.c
    - )
  - )

The above code will generate the output data.frame shown below. The table lists all the sub-regions and their presence across the three sets of region objects (regions.a, regions.b, and regions.c) passed to the function. For instance, sub-region chr1:1-10 (column: index) overlaps with 2 region objects (b and c). This presence is shown as comma separated list in ‘names’ column as well as a truth table in the subsequent columns. The number of columns representing the truth table will match the number of region objects passed to the function bedr.join.multiple.region().

**Table:** 

index n.overlaps names a b c			
1 2 3 4 5 6 7 8 9 10 11 12 13 14 15 16 17 18 19 20 21 22 23 24	chr1:1-10 chr1:10-20 chr1:20-100 chr1:100-101 chr1:101-111 chr1:111-210 chr1:210-211 chr1:211-212 chr1:212-240 chr1:240-250 chr1:2000-2010 chr10:50-100 chr10:100-110 chr10:110-150 chr2:1-5 chr2:5-10 chr2:10-20 chr2:20-30 chr2:30-40 chr2:40-60 chr20:1-5 chr20:6-7 chr20:7-10 chr20:10-12	2 1 2 1 2 3 2 3 2 1 1 1 1 2 2 1 2 1 2 3 1 1 2 1	b,c 0 1 1 a 1 0 0 a,c 1 0 1 c 0 0 1 a,c 1 0 1 a,b,c 1 1 1 b,c 0 1 1 a,b,c 1 1 1 b,c 0 1 1 b 0 1 0 b 0 1 0 a 1 0 0 b 0 1 0 b,c 0 1 1 b,c 0 1 1 c 0 0 1 a,c 1 0 1 a 1 0 0 a,c 1 0 1 a,b,c 1 1 1 a 1 0 0 b 0 1 0 b,c 0 1 1 c 0 0 1

### Subtract and intersect

The subtract utility identifies regions exclusive to first set of regions, and intersect function identifies sub-regions of first set which overlap with the second set of regions (Fig. 2), e.g.

- bedr.subtract.region(
  - x = regions.a,
  - y = regions.b
  - )

The above code will generate the following output which lists the sub-regions exclusive to regions.a:

- "chr1:10-100" "chr10:50-100"
- "chr20:1-5"

Intersect utility makes use of bed.join.region() and finds regions in the second set which overlap with the regions in the first set. An example is shown in the Results section “Join”. Similarly in.region(x = regions.a, y = regions.b) and its R style convenience operator %in.region% can be used to test (logical) presence of overlapping regions, e.g.

- in.region(
  - x = regions.a,
  - y = regions.b
  - )

- FALSE TRUE TRUE FALSE TRUE FALSE

bedr also provides an interface to find overlapping regions using *Tabix* [7]. This can be done using the following bedr call:

- regions.d <- c(
  - "1:1000-100000",
  - "1:1000000-1100000"
  - )
- cosmic.vcf.example <- system.file(
  - "extdata/CosmicCodingMuts_v66_20130725_ex.vcf.gz",
  - package = "bedr"
  - )
- head(
  - tabix(
    - region = regions.d,
    - file.name = cosmic.vcf.example,
    - check.chr = FALSE
    - )
  - )

which identifies regions overlapping with COSMIC coding mutations file resulting in the following *data.frame* (only first six rows are shown below):

**Table:** 

	CHROM	POS	ID	REF	ALT	QUAL	FILTER
1 2 3 4 5 6	1 1 1 1 1 1	69345 69523 69538 69539 69540 69569	COSM911918 COSM426644 COSM75742 COSM1343690 COSM1560546 COSM1599955	C G G T G T	A T A C T C	NA NA NA NA NA NA	<NA> <NA> <NA> <NA> <NA> <NA>

**Table:** 

INFO	
1 2 3 4 5 6	GENE=OR4F5;STRAND=+;CDS=c.255C>A;AA=p.I85I;CNT=1 GENE=OR4F5;STRAND=+;CDS=c.433G>T;AA=p.G145C;CNT=1 GENE=OR4F5;STRAND=+;CDS=c.448G>A;AA=p.V150M;CNT=1 GENE=OR4F5;STRAND=+;CDS=c.449T>C;AA=p.V150A;CNT=1 GENE=OR4F5;STRAND=+;CDS=c.450G>T;AA=p.V150V;CNT=1 GENE=OR4F5;STRAND=+;CDS=c.479T>C;AA=p.L160P;CNT=2

### Third-party compatibility

Given that bedr can process regions data as R’s *vector* as well as *data.frame* data structure, it is easily transformable to other third-party sequence and region objects. For instance, bedr provides a utility adaptor to convert regions into *BED data.frame* as shown below:

- regions.a.bed <- convert2bed(
  - x = regions.a
  - )

which can be further converted to a widely compatible *GRanges* [4] object, as shown below:

- library("GenomicRanges")
- makeGRangesFromDataFrame(
  - df = regions.a.bed
  - )

The above code will create a GRanges object shown in the output below, which can be further customized/extended with additional annotations such as strand and genomic feature names.

**Table:** 

GRanges object with 6 ranges and 0 metadata columns:			
	seqnames	ranges	strand
	<Rle>	<IRanges>	<Rle>
[1] [2] [3] [4] [5] [6]	chr1 chr1 chr1 chr10 chr2 chr20	[10, 100] [101, 210] [211, 212] [50, 100] [10, 60] [1, 5]	* * * * * *
- - - - - - - seqinfo: 4 sequences from an unspecified genome;no seqlengths			

To perform feature meta-analysis and annotation retrieval/conversion (see example workflow in Additional file 1), bedr facilitates downloads from UCSC [8], COSMIC [9] and HUGO [10] including reference genome annotations, repeat sequences, black lists and disease candidate features. Also, bedr has a fully integrated unit-testing framework allowing users to verify integrity of bedr functions when using customized development or installations.

### Visualization

For results of common operations such as intersect, Venn diagrams of overlapping features between 2 to 5 sets of regions (2- to 5-way Venn diagrams) can be generated automatically [11]. The overlap criterion can be defined in a number of ways including unique intervals, gene length or user-specified size as a fraction of sub-region’s length, e.g.

- bedr.plot.region(
  - input = list(
    - a = regions.a,
    - b = regions.b
    - ),
  - feature = "bp",
  - fraction.overlap = 0.1
  - )

The above code will generate a base pair level overlap of sequence objects regions.a and regions.b, and show the results as a Venn diagram highlighting lengths of exclusive and overlapping regions as shown below:

Further, bedr output is ideally suited for alternative complex set visualization tools such as UpSetR [12] and Gviz [13].

## Conclusions

We created bedr; an R package to support genomic operations using the BEDTools [6] and BEDOPS [5] engines. bedr implements an API in R which offers a number of utility functions such as intersect, merge, sort and plotting of genomic intervals as well as provides a unified interface to BEDTools and BEDOPS. These functions are efficient, powerful and perform complex feature annotations and cross-sectional operations on genomic regions. Given that bedr supports two well-established genomic engines, its output is comparable to the native output of these tools, however in R data structures. These features of bedr are urgently needed by bioinformatics research community and will be a timely addition to the catalogue of sequence analysis tools. Further, the interoperability of bedr data structures with *BED* and *GRanges* data.frame/objects makes it an easy-to-fit component in existing genomic pipelines. bedr is freely available as an open-source package through CRAN and lends itself for customized extensions needed for in-house sequencing-analysis pipelines as well as future bioinformatics protocols.

## Availability and requirements

**Project name:** bedr

**Project home page:** http://cran.r-project.org/web/packages/bedr

**Operating system(s):** OSX, Linux/Unix

**Programming language:** R

**Other requirements:** BEDTools, BEDOPS

**License:** e.g. GNU GPL-2

**Any restrictions to use by non-academics:** None

## Additional file

Additional file 1: Example workflow. (DOCX 30 kb)

## Abbreviations

API: Application programming interface
BED: Browser extensible display
chr: Chromosome
COSMIC: Catalogue of somatic mutations in cancer
CRAN: The comprehensive R archive network
DAS: Distributed annotation system
GFF: General feature format
GTF: Gene transfer format
HUGO: Human Genome Organisation
VCF: Variant call format
