# Erratum to: A bedr way of genomic interval processing

**DOI:** 10.1186/s13029-016-0061-y

**Published:** 2017-01-09

**Authors:** Syed Haider, Daryl Waggott, Emilie Lalonde, Clement Fung, Fei-Fei Liu, Paul C. Boutros

**Affiliations:** 1 Informatics and Biocomputing Platform, Ontario Institute for Cancer Research, Toronto, M5G 0A3 Canada; 2 Departments of Radiation Oncology, Pharmacology & Toxicology, and Medical Biophysics, University of Toronto, Toronto, M5G 2M9 Canada; 3 Ontario Cancer Institute and Campbell Family Institute for Cancer Research, Princess Margaret Hospital, University Health Network, Toronto, M5G 2M9 Canada

## Erratum

In the HTML version of this article that was originally published [1] the Venn diagram in the section titled “Visualization” was missing and was replaced with source code. Please see the missing Venn diagram below:
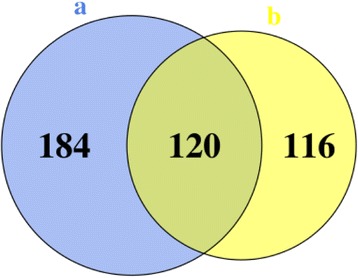


The original article has been revised and the publisher apologises for the error.
