# Supplementary material for: A bedr way of genomic interval processing
Source: Source Code Biol Med. 2016 Dec 15;11:14. doi: 10.1186/s13029-016-0059-5 (PMC5157088; doi:10.1186/s13029-016-0059-5)
Supplement: Additional file 1: — Example workflow. (DOCX 30 kb) [file 13029_2016_59_MOESM1_ESM.docx]

# A bedr way of genomic interval processing

Syed Haider^1,+^, Daryl Waggott^1,+^, Emilie Lalonde^1,2^_,_ Clement Fung^1^, Fei-Fei Liu^2,3^, Paul C. Boutros^1,2,*^

^1^ Informatics and Biocomputing Platform, Ontario Institute for Cancer Research, Toronto, Canada, M5G 0A3

^2^ Departments of Radiation Oncology, Pharmacology & Toxicology, and Medical Biophysics, University of Toronto, Toronto, Canada, M5G 2M9

^3^ Ontario Cancer Institute and Campbell Family Institute for Cancer Research, Princess Margaret Hospital, University Health Network, Toronto, Canada, M5G 2M9

+ Equal contributions

* Corresponding author

Emails:

Syed Haider: Syed.Haider@oicr.on.ca

Daryl Waggott: dwaggott@gmail.com

Emilie Lalonde: Emilie.Lalonde@oicr.on.ca

Clement Fung: clement.y.fung@gmail.com

Fei-Fei Liu: Fei-Fei.Liu@rmp.uhn.on.ca

Paul C. Boutros: Paul.Boutros@oicr.on.ca

**Example Workflow**

The example workflow below reads two VCF files from different variant callers, further limiting to structural variants only and finally identifying common calls. This workflow annotates the structural variants with RefSeq gene identifiers if the variant overlaps with the gene body for instance, further limiting to chromosome 1 and 2 only. Further, it collapses multiple gene entries into a single entry (row).

# initialize column headers and vcf file names

VALID.SV.TYPES **<-** c('BND', 'CNV', 'DEL', 'DUP', 'INS', 'INV');

POSITION.COLUMNS **<-** c('CHROM', 'POS', 'END');

callerA.filename **<-** system.file("extdata/callerA.vcf.gz", package = "bedr");

callerB.filename **<-** system.file("extdata/callerB.vcf.gz", package = "bedr");

# read the VCF file

callerA **<-** read.vcf(callerA.filename, split.info = TRUE, verbose = TRUE)$vcf;

callerB **<-** read.vcf(callerB.filename, split.info = TRUE, verbose = TRUE)$vcf;

# focus on SVs

callerA **<-** callerA[which(callerA$SVTYPE %in% VALID.SV.TYPES), ];

callerB **<-** callerB[which(callerB$SVTYPE %in% VALID.SV.TYPES), ];

# convert to zero-based coordinates

callerA$POS **<-** callerA$POS - 1;

callerB$POS **<-** callerB$POS - 1;

# find all overlapping pairs, retrieve size of overlap (bp)

overlapping.pairs **<-** bedr.join.region(

    callerA[, POSITION.COLUMNS],

    callerB[, POSITION.COLUMNS],

    report.n.overlap = TRUE,

    check.chr = FALSE,

verbose = TRUE

    );

colnames(overlapping.pairs) **<-** c(

    'a.CHROM', 'a.POS', 'a.END',

    'b.CHROM', 'b.POS', 'b.END',

    'Overlap'

    );

overlapping.pairs$b.POS **<-** as.numeric(overlapping.pairs$b.POS);

overlapping.pairs$b.END **<-** as.numeric(overlapping.pairs$b.END);

# compute a distance between overlapping pairs

min.breakpoint.distances **<-** cbind(

    overlapping.pairs$a.POS - overlapping.pairs$b.POS,

    overlapping.pairs$a.END - overlapping.pairs$b.END

    );

min.breakpoint.distances **<-** apply(

    abs(min.breakpoint.distances),

    1,

    min

    );

a.length **<-** overlapping.pairs$a.END - overlapping.pairs$a.POS;

b.length **<-** overlapping.pairs$b.END - overlapping.pairs$b.POS;

overlapping.pairs$distance  **<-** (min.breakpoint.distances + abs(a.length - b.length)) / 2;

# print

cat(" OVERLAPPING PAIRS: \n"); print(head(overlapping.pairs)); cat("\n");

The code above will generate the following verbose messages and output. Log info can be controlled using logical parameter *verbose* = [TRUE|FALSE] in all bedr methods. The genomic coordinates and length of overlapping regions between two variant callers are displayed in each row:

## * Checking path for bedtools... PASS

## /Users/SW/bedtools2/bin/bedtools

## READING VCF

## * checking if file exists... PASS

## * Reading vcf header...

## Done

## * Reading vcf body...

## Done

## * Parse vcf header...

## Done

## * Split info...

## * Done

## READING VCF

## * checking if file exists... PASS

## * Reading vcf header...

## Done

## * Reading vcf body...

## Done

## * Parse vcf header...

## Done

## * Split info...

## * Done

## JOINING

## * Processing input (1): a

## CONVERT TO BED

## * Checking input type... PASS

## Input seems to be in bed format but chr/start/end column names are missing

## VALIDATE REGIONS

## * Check if index is a string... PASS

## * Check index pattern... PASS

## * Check for missing values... PASS

## * Check for larger start position... PASS.

## * Check if zero based... PASS

## * Checking sort order... PASS

## * Checking for overlapping 'contiguous' regions... PASS

## * Processing input (2): b

## CONVERT TO BED

## * Checking input type... PASS

## Input seems to be in bed format but chr/start/end column names are missing

## VALIDATE REGIONS

## * Check if index is a string... PASS

## * Check index pattern... PASS

## * Check for missing values... PASS

## * Check for larger start position... PASS.

## * Check if zero based... PASS

## * Checking sort order... PASS

## * Checking for overlapping 'contiguous' regions... PASS

## bedtools intersect -a /var/folders/ty/msz_v3ln0vs_480xb6hflwqw0000gn/T//Rtmpm4692t/a_b34734f42df.bed -b /var/folders/ty/msz_v3ln0vs_480xb6hflwqw0000gn/T//Rtmpm4692t/b_b3477f06b4f7.bed -wo -sorted

## OVERLAPPING PAIRS:

## a.CHROM a.POS a.END b.CHROM b.POS b.END Overlap distance

## 1 1 247204800 247209976 1 247205240 247209984 4736 220.0

## 2 1 247874052 247886918 1 247874057 247877965 3908 4481.5

## 3 10 5304636 5309227 10 5202123 5324657 4591 66686.5

## 4 10 12504806 12506154 10 12504806 12506113 1307 20.5

## 5 10 19431941 19433784 10 19431472 19434296 1843 725.0

## 6 10 36026185 36030048 10 36026100 36030053 3863 47.5

Next, read the RefSeq data to annotate the structural variants (SVs) with gene identifiers:

# get Human RefSeq genes (Hg19) in BED format

refseq.file **<-** system.file("extdata/ucsc.hg19.RefSeq.chr1-2.txt.gz", package = "bedr");

refseq **<-** read.table(refseq.file, header = FALSE, stringsAsFactors = FALSE);

# sort Refseq and remove chr prefix

refseq.sorted **<-** bedr.sort.region(refseq[, 1:4], verbose = TRUE);

colnames(refseq.sorted) **<-** c(POSITION.COLUMNS, "Gene");

refseq.sorted$CHROM **<-** gsub("^chr", "", refseq.sorted$CHROM);

# add gene identifiers to callerA

callerA.annotated **<-** bedr.join.region(

    callerA[, POSITION.COLUMNS],

    refseq.sorted,

    report.n.overlap = TRUE,

    check.chr = FALSE,

verbose = TRUE

    );

colnames(callerA.annotated) **<-** c(

    'a.CHROM', 'a.POS', 'a.END',

    'Gene.CHROM', 'Gene.POS', 'Gene.END', 'Gene', 'Overlap'

    );

# add gene identifiers to callerB

callerB.annotated **<-** bedr.join.region(

    callerB[, POSITION.COLUMNS],

    refseq.sorted,

    report.n.overlap = TRUE,

    check.chr = FALSE,

verbose = TRUE

    );

colnames(callerB.annotated) **<-** c(

    'b.CHROM', 'b.POS', 'b.END',

    'Gene.CHROM', 'Gene.POS', 'Gene.END', 'Gene', 'Overlap'

    );

# reinstate chr prefix to chromosome names

callerA.annotated$a.CHROM **<-** paste('chr', callerA.annotated$a.CHROM, sep = "");

callerB.annotated$b.CHROM **<-** paste('chr', callerB.annotated$b.CHROM, sep = "");

# print

cat(" CALLER A GENES (chr 1,2): \n"); print(head(callerA.annotated)); cat("\n");

cat(" CALLER B GENES (chr 1,2): \n"); print(head(callerB.annotated)); cat("\n");

The code above will generate the following verbose messages and output, with gene coordinates and identifiers appended as new columns to each of the variant callers’ data:

## * Checking path for bedtools... PASS

## /Users/SW/bedtools2/bin/bedtools

## SORTING

## VALIDATE REGIONS

## * Checking input type... PASS

## Input seems to be in bed format but chr/start/end column names are missing

## * Check if index is a string... PASS

## * Check index pattern... PASS

## * Check for missing values... PASS

## * Check for larger start position... PASS.

## * Check if zero based... PASS

## JOINING

## * Processing input (1): a

## CONVERT TO BED

## * Checking input type... PASS

## Input seems to be in bed format but chr/start/end column names are missing

## VALIDATE REGIONS

## * Check if index is a string... PASS

## * Check index pattern... PASS

## * Check for missing values... PASS

## * Check for larger start position... PASS.

## * Check if zero based... PASS

## * Checking sort order... PASS

## * Checking for overlapping 'contiguous' regions... PASS

## * Processing input (2): b

## CONVERT TO BED

## * Checking input type... PASS

## Input seems to be in bed format but chr/start/end column names are missing

## VALIDATE REGIONS

## * Check if index is a string... PASS

## * Check index pattern... PASS

## * Check for missing values... PASS

## * Check for larger start position... PASS.

## * Check if zero based... PASS

## * Checking sort order... FAIL

## The input for object is not *lexographically* ordered!

## This can cause unexpected results for some set operations.

## try: x <- bedr.sort.region(x)

## * Checking for overlapping 'contiguous' regions... FAIL

## The input for object has overlapping features!

## This can cause unexpected results for some set operations.

## i.e. x <- bedr.merge.region(x)

## bedtools intersect -a /var/folders/ty/msz_v3ln0vs_480xb6hflwqw0000gn/T//Rtmpm4692t/a_b3474fb85d3f.bed -b /var/folders/ty/msz_v3ln0vs_480xb6hflwqw0000gn/T//Rtmpm4692t/b_b34776330992.bed -wo -sorted

## JOINING

## * Processing input (1): a

## CONVERT TO BED

## * Checking input type... PASS

## Input seems to be in bed format but chr/start/end column names are missing

## VALIDATE REGIONS

## * Check if index is a string... PASS

## * Check index pattern... PASS

## * Check for missing values... PASS

## * Check for larger start position... PASS.

## * Check if zero based... PASS

## * Checking sort order... PASS

## * Checking for overlapping 'contiguous' regions... PASS

## * Processing input (2): b

## CONVERT TO BED

## * Checking input type... PASS

## Input seems to be in bed format but chr/start/end column names are missing

## VALIDATE REGIONS

## * Check if index is a string... PASS

## * Check index pattern... PASS

## * Check for missing values... PASS

## * Check for larger start position... PASS.

## * Check if zero based... PASS

## * Checking sort order... FAIL

## The input for object is not *lexographically* ordered!

## This can cause unexpected results for some set operations.

## try: x <- bedr.sort.region(x)

## * Checking for overlapping 'contiguous' regions... FAIL

## The input for object has overlapping features!

## This can cause unexpected results for some set operations.

## i.e. x <- bedr.merge.region(x)

## bedtools intersect -a /var/folders/ty/msz_v3ln0vs_480xb6hflwqw0000gn/T//Rtmpm4692t/a_b3476c5f1884.bed -b /var/folders/ty/msz_v3ln0vs_480xb6hflwqw0000gn/T//Rtmpm4692t/b_b34719534c94.bed -wo -sorted

## CALLER A GENES (chr 1,2):

## a.CHROM a.POS a.END Gene.CHROM Gene.POS Gene.END Gene

## 1 chr1 32176186 32193834 1 32192705 32229664 NM_001294336

## 2 chr1 32176186 32193834 1 32192705 32229664 NM_001294335

## 3 chr1 36419037 36421454 1 36396682 36522063 NM_024852

## 4 chr1 36419037 36421454 1 36396682 36522063 NM_177422

## 5 chr1 40302119 40310286 1 40306705 40349177 NM_017646

## 6 chr1 52748187 52752298 1 52607765 52812358 NM_007324

## Overlap

## 1 1129

## 2 1129

## 3 2417

## 4 2417

## 5 3581

## 6 4111

##

## CALLER B GENES (chr 1,2):

## b.CHROM b.POS b.END Gene.CHROM Gene.POS Gene.END Gene

## 1 chr1 235776110 235776340 1 235676124 235881413 NR_039973

## 2 chr1 235776110 235776340 1 235710984 235813293 NM_001098722

## 3 chr1 235776110 235776340 1 235710984 235814054 NM_001098721

## 4 chr1 235776110 235776340 1 235710984 235814054 NM_004485

## 5 chr1 236025376 236025711 1 235824330 236030227 NM_000081

## 6 chr1 236025376 236025711 1 235824330 236047008 NM_001301365

## Overlap

## 1 230

## 2 230

## 3 230

## 4 230

## 5 335

## 6 335

Finally, to collapse multiple gene entries in one row. This can be done using bedtools groupby utility as shown below:

# collapse column 7 (Gene) against unique composite key (column 1, 2 and 3)

callerA.annotated.grouped **<-** bedr(

    input = list(i = callerA.annotated),

    method = "groupby",

    params = "-g 1,2,3 -c 7 -o collapse",

verbose = TRUE

    );

# collapse column 7 (Gene) against unique composite key (column 1, 2 and 3)

callerB.annotated.grouped **<-** bedr(

    input = list(i = callerB.annotated),

    method = "groupby",

    params = "-g 1,2,3 -c 7 -o collapse",

verbose = TRUE

    );

# print

cat(" CALLER A GENES (chr 1,2) GROUPED: \n"); print(head(callerA.annotated.grouped)); cat("\n");

cat(" CALLER B GENES (chr 1,2) GROUPED: \n"); print(head(callerB.annotated.grouped)); cat("\n");

The code above will generate the following verbose messages and output, where multiple genes spanning the same SV region are collapsed in the last column for both SV callers:

## * Checking path for bedtools... PASS

## /Users/shaider/Desktop/phd_work/SW/bedtools2/bin/bedtools

## * Processing input (1): i

## CONVERT TO BED

## * Checking input type... PASS

## Input seems to be in bed format but chr/start/end column names are missing

## VALIDATE REGIONS

## * Check if index is a string... PASS

## * Check index pattern... PASS

## * Check for missing values... PASS

## * Check for larger start position... PASS.

## * Check if zero based... PASS

## * Checking sort order... FAIL

## The input for object is not *lexographically* ordered!

## This can cause unexpected results for some set operations.

## try: x <- bedr.sort.region(x)

## * Checking for overlapping 'contiguous' regions... FAIL

## The input for object has overlapping features!

## This can cause unexpected results for some set operations.

## i.e. x <- bedr.merge.region(x)

## bedtools groupby -i /var/folders/ty/msz_v3ln0vs_480xb6hflwqw0000gn/T//Rtmpm4692t/i_b3474783112d.bed -g 1,2,3 -c 7 -o collapse

## * Processing input (1): i

## CONVERT TO BED

## * Checking input type... PASS

## Input seems to be in bed format but chr/start/end column names are missing

## VALIDATE REGIONS

## * Check if index is a string... PASS

## * Check index pattern... PASS

## * Check for missing values... PASS

## * Check for larger start position... PASS.

## * Check if zero based... PASS

## * Checking sort order... FAIL

## The input for object is not *lexographically* ordered!

## This can cause unexpected results for some set operations.

## try: x <- bedr.sort.region(x)

## * Checking for overlapping 'contiguous' regions... FAIL

## The input for object has overlapping features!

## This can cause unexpected results for some set operations.

## i.e. x <- bedr.merge.region(x)

## bedtools groupby -i /var/folders/ty/msz_v3ln0vs_480xb6hflwqw0000gn/T//Rtmpm4692t/i_b347ad7b5fb.bed -g 1,2,3 -c 7 -o collapse

## CALLER A GENES (chr 1,2) GROUPED:

## V1 V2 V3 V4

## chr1:32176186-32193834 chr1 32176186 32193834 NM_001294336,NM_001294335

## chr1:36419037-36421454 chr1 36419037 36421454 NM_024852,NM_177422

## chr1:40302119-40310286 chr1 40302119 40310286 NM_017646

## chr1:52748187-52752298 chr1 52748187 52752298 NM_007324,NM_004799

## chr1:62421145-62424426 chr1 62421145 62424426 NM_176877

## chr1:67068898-67075815 chr1 67068898 67075815 NM_001308203,NM_032291

##

## CALLER B GENES (chr 1,2) GROUPED:

## V1 V2 V3

## chr1:235776110-235776340 chr1 235776110 235776340

## chr1:236025376-236025711 chr1 236025376 236025711

## chr1:236225022-236225405 chr1 236225022 236225405

## chr1:236440947-236441376 chr1 236440947 236441376

## chr1:236594960-236595252 chr1 236594960 236595252

## chr1:236882374-236882744 chr1 236882374 236882744

## V4

## chr1:235776110-235776340 NR_039973,NM_001098722,NM_001098721,NM_004485

## chr1:236025376-236025711 NM_000081,NM_001301365,NR_102436

## chr1:236225022-236225405 NM_002508

## chr1:236440947-236441376 NM_019891

## chr1:236594960-236595252 NM_145861,NM_080738

## chr1:236882374-236882744 NM_001278343,NM_001278344,NM_001103

N.B. Log info printed through `verbose = T` should be carefully assessed. Often, status=FAIL may just highlight a potential problem, and hence code continues to execute without a graceful failure. Therefore, please do read the log messages carefully.

For further usage examples and workflows, please see bedr package on CRAN:

https://cran.r-project.org/package=bedr
